# Supplementary material for: Human genetic analyses of organelles highlight the nucleus in age-related trait heritability
Source: eLife. 2021 Sep 1;10:e68610. doi: 10.7554/eLife.68610 (PMC8476128; doi:10.7554/eLife.68610)
Supplement: Supplementary file 1. [file elife-68610-supp1.pdf]

		UK Biobank						Meta analysis					
Phenotype	Median onset age	Code	N	Cases	Controls	h^2	h^2_{se}	Study	N	Cases	Controls	h^2	h^2_{se}
GERD	50	K21	361194	10743	350451	0.08	0.01						
Deafness	52	3393	219358	10942	208416	0.10	0.02						
Raised Total Cholesterol	56	20002_1473	361141	43957	317184	0.12	0.01						
HDL-C	57†	30760_irnt	315133	–	–	0.22	0.01	Teslovich et al., 2010	99900*	–	–	0.12	0.01
LDL-C	57†	30780_irnt	343621	–	–	0.10	0.01	Teslovich et al., 2010	95454*	–	–	0.10	0.01
Triglycerides	57†	30870_irnt	343992	–	–	0.17	0.01	Teslovich et al., 2010	96598*	–	–	0.12	0.02
Diastolic BP	59†	4079_irnt	340162	–	–	0.14	0.01	ICBP 2011	69395*	–	–	0.14	0.02
Hypertension	59	20002_1065	361141	93560	267581	0.22	0.01						
Systolic BP	59†	4080_irnt	340159	–	–	0.15	0.01	ICBP 2011	69395*	–	–	0.14	0.02
Diaphragmatic Hernia	60	K44	361194	8042	353152	0.08	0.02						
Glucose	61†	30740_irnt	314916	–	–	0.08	0.01	Manning et al. 2012	58074	–	–	0.09	0.02
Osteoarthritis	61	20002_1465	361141	30046	331095	0.07	0.01						
Type 2 Diabetes Mellitus	61	2443	360192	17275	342917	0.21	0.02	Morris et al. 2012	69033*	12171*	56862*	0.18	0.03
Angina	64	20002_1074	361141	11370	349771	0.17	0.02						
Myocardial Infarction	65	I9_CHD	361194	10157	351037	0.14	0.02						
Diverticular Disease	67	K57	361194	12662	348532	0.13	0.01						
Primary Skin Malignancy	68	C3_SKIN	361194	16531	344663	0.16	0.03						
Coronary Artery Disease	70	I9_IHD	361194	20857	340337	0.15	0.01	Schunkert et al., 2011	86995*	22233*	64762*	0.07	0.01
Osteoporosis/BMD	71†	3148_irnt	206496	–	–	0.28	0.02	Estrada et al 2012	32961	–	–	0.26	0.03
Atrial Fibrillation	74	I48	361194	6356	354838	0.14	0.03	Christophersen et al. 2017	133073	17931	115142	0.06	0.01
Cataracts	74	H26	361194	11306	349888	0.09	0.01						
eGFR	74†							Pattaro et al., 2016	133413*	–	–	0.12	0.01
Parkinson's Disease§	74							Nalls et al., 2019	482730**	33674**	449056**	0.03	0.00
Alzheimer's Disease	83							Lambert et al., 2013	54162	17008	37154	0.05	0.01

We only show sample sizes for traits with heritability Z score ≥ 4 as obtained by S-LDSC.
* variant-specific sample sizes used in analysis; displayed values are maximal values reported in associated publication
** variant-specific sample size used in analysis and to obtain displayed maximal values
§ meta-analysis contains samples also part of UKB
† median age of onset for continuous traits obtained from discretizing distribution by Kuan et al. or from surrogate diseased states (T2DM for Glucose; CKD for eGFR)
