# Supplementary material for: Human genetic analyses of organelles highlight the nucleus in age-related trait heritability
Source: eLife. 2021 Sep 1;10:e68610. doi: 10.7554/eLife.68610 (PMC8476128; doi:10.7554/eLife.68610)
Supplement: Transparent reporting form [file elife-68610-transrepform.pdf]

Westbrook Centre
Milton Road
Cambridge CB4 1YG, UK

P 01223 855340
W elifesciences.org
T @elife

eLife's transparent reporting form

We encourage authors to provide detailed information *within their submission* to facilitate the interpretation and replication of experiments. Authors can upload supporting documentation to indicate the use of appropriate reporting guidelines for health-related research (see EQUATOR Network), life science research (see the BioSharing Information Resource), or the ARRIVE guidelines for reporting work involving animal research. Where applicable, authors should refer to any relevant reporting standards documents in this form.

If you have any questions, please consult our Journal Policies and/or contact us: editorial@elifesciences.org.

Sample-size estimation

- You should state whether an appropriate sample size was computed when the study was being designed
- You should state the statistical method of sample size computation and any required assumptions
- If no explicit power analysis was used, you should describe how you decided what sample (replicate) size (number) to use

Please outline where this information can be found within the submission (e.g., sections or figure legends), or explain why this information doesn't apply to your submission:

> Sample size was not predetermined, as we used the maximal number of samples available in the UK Biobank after sample QC. We performed empirical power analysis for gene-based enrichment analyses (figure 2-figure supplement 6) to show that with the included sample sizes, we have acceptable power for detection of physiologically relevant enrichment effect sizes across our selected traits. Further, we used positive controls (e.g., tissue expressed gene-sets, creatinine and aspartate aminotransferase which showed associations with mtDNA in previous work) to ensure that previously detected associations and enrichments were detectable with our sample size. These are described in detail in the text, Materials and Methods, and in the Appendix.

Replicates

- You should report how often each experiment was performed
- You should include a definition of biological versus technical replication
- The data obtained should be provided and sufficient information should be provided to indicate the number of independent biological and/or technical replicates
- If you encountered any outliers, you should describe how these were handled
- Criteria for exclusion/inclusion of data should be clearly stated
- High-throughput sequence data should be uploaded before submission, with a private link for reviewers provided (these are available from both GEO and ArrayExpress)

Please outline where this information can be found within the submission (e.g., sections or figure legends), or explain why this information doesn't apply to your submission:

eLife Sciences Publications, Ltd is a limited liability non-profit non-stock corporation incorporated in the State of Delaware, USA, with company number 5030732, and is registered in the UK with company number FC030576 and branch number BR015634 at the address Westbrook Centre, Milton Road, Cambridge CB4 1YG.

As mentioned in the main text, to replicate our results, we collated GWAS meta-analyses from the literature with cohorts that were non-overlapping with the discovery cohort (UKB). Of our 21 UKB traits, we identified 10 with well-powered (albeit reduced relative to UKB) replication cohorts. We replicated the lack of enrichment for mitochondrial genes for all 10 cohorts. In parallel, we showed a lack of enrichment for mitochondrial genes in GWAS Catalog for all tested traits. Our observed enrichment among transcription factors for several diseases in UKB replicated among meta-analyses for many (but not all) traits. Any discordance is likely attributable to reduced power among the meta-analyses compared to UKB. We also used independent methods (S-LDSC and MAGMA) and found concordance of our results between the two approaches.

eLife Sciences Publications, Ltd is a limited liability non-profit non-stock corporation incorporated in the State of Delaware, USA, with company number 5030732, and is registered in the UK with company number FC030576 and branch number BR015634 at the address Westbrook Centre, Milton Road, Cambridge CB4 1YG.

Statistical reporting

- Statistical analysis methods should be described and justified
- Raw data should be presented in figures whenever informative to do so (typically when N per group is less than 10)
- For each experiment, you should identify the statistical tests used, exact values of N, definitions of center, methods of multiple test correction, and dispersion and precision measures (e.g., mean, median, SD, SEM, confidence intervals; and, for the major substantive results, a measure of effect size (e.g., Pearson's r, Cohen's d)
- Report exact p-values wherever possible alongside the summary statistics and 95% confidence intervals. These should be reported for all key questions and not only when the p-value is less than 0.05.

Please outline where this information can be found within the submission (e.g., sections or figure legends), or explain why this information doesn't apply to your submission:

Statistical methods and multiple testing correction approaches are described in detail in the Materials and Methods, in brief in figure legends and main text. Point estimates are reported alongside p-values for all enrichment tests, and individual points are shown when informative (e.g., individual associations for mtDNA-GWAS). Error bars are defined in figure legends and typically represent 95% CI. -log10 p-values when plotted are accompanied by raw values in the supplementary tables. Sample size information is provided in the main text and in supplementary file 1.

(For large datasets, or papers with a very large number of statistical tests, you may upload a single table file with tests, Ns, etc., with reference to sections in the manuscript.)

Group allocation

- Indicate how samples were allocated into experimental groups (in the case of clinical studies, please specify allocation to treatment method); if randomization was used, please also state if restricted randomization was applied
- Indicate if masking was used during group allocation, data collection and/or data analysis

Please outline where this information can be found within the submission (e.g., sections or figure legends), or explain why this information doesn't apply to your submission:

We in large part use summary statistics from previously performed GWAS on either UKB or other large cohorts, and thus do not directly allocate samples into experimental groups. For mtDNA-GWAS, phenotype definitions were used as provided by the UK Biobank resource and as used for the Neale Lab UKB Round 2 GWAS.

Additional data files ("source data")

- We encourage you to upload relevant additional data files, such as numerical data that are represented as a graph in a figure, or as a summary table
- Where provided, these should be in the most useful format, and they can be uploaded as "Source data" files linked to a main figure or table
- Include model definition files including the full list of parameters used
- Include code used for data analysis (e.g., R, MatLab)
- Avoid stating that data files are "available upon request"

Please indicate the figures or tables for which source data files have been provided:

eLife Sciences Publications, Ltd is a limited liability non-profit non-stock corporation incorporated in the State of Delaware, USA, with company number 5030732, and is registered in the UK with company number FC030576 and branch number BR015634 at the address Westbrook Centre, Milton Road, Cambridge CB4 1YG.

Genetic and phenotypic correlation point estimates and standard errors/p-values plotted in Figure 1B are available in Figure 1-source data 1. Summary statistics from mtDNA-GWAS (Figure 2C, Figure 2-figure supplement 9) are available in Source data 2. All gene-based enrichment analysis p-values and point estimates (corresponding to figures 2B, 2D, figure 2-figure supplement 2–figure 2-figure supplement 5, figure 2-figure supplement 7, figure 3, figure 3-figure supplement 2–figure 3-figure supplement 8, figure 4, figure 4-figure supplement 1) are available in Source data 1 and Source data 3.

eLife Sciences Publications, Ltd is a limited liability non-profit non-stock corporation incorporated in the State of Delaware, USA, with company number 5030732, and is registered in the UK with company number FC030576 and branch number BR015634 at the address Westbrook Centre, Milton Road, Cambridge CB4 1YG.
